# Cardiac Muscle Training—A New Way of Recognizing and Supporting Recovery for LVAD Patients in the Pediatric Population

**DOI:** 10.3390/life12111681

**Published:** 2022-10-22

**Authors:** Anca Racolta, Jae-Hyun Johannes Ahn, Marinos Kantzis, Hendrik Milting, Volker Lauenroth, Hermann Körperich, Eugen Sandica, Stephan Schubert, Kai Thorsten Laser

**Affiliations:** 1 Clinic for Pediatric Cardiology, Center for Congenital Heart Defects, University Hospital RWTH Aachen, 52074 Aachen, Germany; 2 Pediatric Heart Center and Center for Congenital Heart Defects, Heart and Diabetes Center NRW, University Hospitals Ruhr University of Bochum, 32545 Bad Oeynhausen, Germany; 3 University Hospital Leicester, NHS Trust EMCHC, Leicester LE1 5WW, UK; 4 Erich and Hanna Klessmann Institute, Heart and Diabetes Center NRW, University Hospitals Ruhr University of Bochum, 32545 Bad Oeynhausen, Germany; 5 Clinic for Thoracic and Cardiovascular Surgery, Heart and Diabetes Center NRW, University Hospitals Ruhr University of Bochum, 32545 Bad Oeynhausen, Germany; 6 Institute for Radiology, Nuclear Medicine and Molecular Imaging, Heart and Diabetes Center NRW, University Hospitals Ruhr University of Bochum, 32545 Bad Oeynhausen, Germany

**Keywords:** congestive heart failure, dilatative cardiomyopathy, assist device, Berlin Heart, weaning, myocarditis

## Abstract

Patients with refractory heart failure due to chronic progressive cardiac myopathy (CM) may require mechanical circulatory support as a bridge to transplantation. A few patients can be weaned from support devices if recovery can be achieved. The identification of these patients is of great importance as recovery may be missed if the heart is unloaded by the ventricular assist device (VAD). Testing the load-bearing capacity of the supported left ventricle (LV) by temporarily and gradually reducing mechanical support during cardiac exercise can help identify responders and potentially aid the recovery process. An exercise training protocol was used in 3 patients (8 months, 18 months and 8 years old) with histological CM findings and myocarditis. They were monitored regularly using clinical information and functional imaging with VAD support. Echocardiographic examination included both conventional real-time 3D echocardiography (RT3DE) and speckle tracking (ST). A daily temporary reduction in pump rate (phase A) was followed by a permanent reduction in rate (phase B). Finally, pump stops of up to 30 min were performed once a week (phase C). The final decision on explantation was based on at least three pump stops. Two patients were weaned and successfully removed from the VAD. One of them was diagnosed with acute viral myocarditis. The other had chronic myocarditis with dilated myopathy and mild interstitial fibrosis. The noninvasive assessment of cardiac output and strain under different loading conditions during VAD therapy is feasible and helps identify candidates for weaning despite severe histological findings. The presented protocol, which incorporates new echocardiographic techniques for determining volume and deformation, can be of great help in positively guiding the process of individual recovery, which may be essential for selecting and increasing the number of patients to be weaned from VAD.

## 1. Introduction

This work is based on the hypothesis that in some carefully selected cases, the myocardium can be trained by gradually offering more volume and reloading the left ventricle. Using a new weaning protocol, a consistent progressive reloading of the ventricle was performed and adapted to the patient’s needs. This means that the time frame of the unloading steps can be changed and adapted to the needs of the patient according to the functional echocardiographic data. The entire procedure can be considered analogous to a hypertrophy-oriented resistance training program. The indication for the implantation of a VAD in the paediatric population is end-stage heart failure despite drug treatment. It usually occurs due to an acute trigger (e.g., acute myocarditis), chronic progressive cardiomyopathy or congenital heart defect (surgically treated or not). The VAD is used in these cases as a bridge until recovery or transplantation. The Berlin Heart Excor is a pulsatile circulatory support system used for paediatric heart failure. The Excor pump offers different ventricle sizes to meet the cardiac output needs of newborns, infants and children. The ventricles are extracorporeal, allowing inspection to rule out clots, look for membrane defects or simply check the filling status of the chambers. There are institutional differences in terms of venous management, anticoagulation drug therapy and maintenance, resulting in a variety of clinical outcomes [1,2,3].

## 2. Methods

### 2.1. General Facts

Between April 2016 and March 2018, 3 patients (8 months, 18 months and 8 years old) were studied as part of the implementation of this method for weaning from a pulsatile LVAD. The patients were admitted to our department for acute heart failure and a ventricular assist device (VAD) was decided as bridging therapy.

The left ventricular support consisted of a pulsatile system, namely a Berlin Heart Excor VAD (Berlin heart AG, Berlin, Germany), with a 15-mL ventricle and, in the case of the older child, a 30-mL ventricle, which was reduced to a 15-mL ventricle shortly after implantation. Endomyocardial biopsy (EMB) samples were taken intraoperatively. The EMB samples were examined at the Institute of Pathology and Neuropathology, Department of General and Molecular Pathology and Pathological Anatomy, University of Tübingen, Germany. Metabolic diseases and coronary anomalies were excluded in all patients. Each patient underwent a standard laboratory procedure on admission. The baseline laboratory tests on admission included blood count; renal (e.g., creatinine) and liver parameters; C-reactive protein; troponin; brain natriuretic peptide (BNP), autoimmune antibodies and extended infectious diagnostics including adenoviruses, echoviruses, enteroviruses, herpes viruses (CMV, EBV, HHV6), hepatitis C, varicella, influenza type A, parainfluenza and parvovirus B19, toxoplasma and borellia. Trends in troponin, BNP and end-organ parameters such as creatinine were included in decision making during the weaning process.

The cornerstone for the weaning decision was the summation of multiple echocardiographic data. In addition to conventional echocardiographic methods, RT3DE and deformation imaging were used. Patients were studied according to a standard protocol for data collection published elsewhere [4,5,6,7,8]. Percentiles for 2D data were based on the publication by Petterson [9]. A Vivid E9 (GE Medical Systems, Milwaukee, WI, USA, SCh, for speckle tracking) with standard probes and an IE33 (Phillips Medical Systems, Best, The Netherlands for the RT3DE, X5-1 transducer) ultrasound unit were used. Data were calculated using LV-Analysis 3.1 (Tomtec, Unterschleissheim, Germany) for RT3DE and Echo Pac version 6.1.2 (GE Medical System) for speckle tracking. The echodiographic data were correlated with the results of several blood gas analyses (ABG) and laboratory data, especially BNP and troponin values.

### 2.2. Weaning Protocol

The protocol consists of three different phases. In the first phase, the frequency of the VAD was reduced daily by 20–25% up to 4 h per day. If echocardiographic parameters were stable, the next phase was initiated with a definitive reduction in frequency to the minimum allowable pump rate of 50–60 beats per minute (bpm). The third and decisive phase was analogous to the off-pump trials in adult cardiology. Our pump stops lasted up to 30 min once a week, with an additional heparin bolus of 100 IU/kg/dose.

During the weaning protocol, we collected clinical, laboratory (ABG, BNP, troponin, end-organ parameters, e.g., liver and kidney) and 2D echocardiographic data, as well as speckle tracking (ST), VTI measurements and RT3DE recordings of left ventricular enddiastolic (LVEDV) and endsystolic (LVESV), stroke volume (SV) and ejection fraction (EF). The haemodynamic parameters were confirmed in the catheter laboratory.

### 2.3. Weaning Protocol Overview

Patient 1 was weaned over 14 weeks. The program included a daily reduction of the frequency for 2 h in the first phase, then an escalation up to 4 h per day and finally a complete reduction of the basic frequency. In the last phase, a stress test in the form of a pump stop was performed.

Patient 2 was weaned in the first phase over 19 weeks by reducing the frequency daily to 10% of the baseline frequency. If the results were promising, a fixed frequency reduction was applied in the second phase, as well as a daily frequency reduction of 10% limited to four hours. The last phase was based on a pump output of only 1.8 L/m^2^/min, followed by a pump stop test.

Patient 3 was implanted with a 30 mL Berlin Heart Ventricle that had to be downsized after only 2 weeks due to rapid recovery and filling problems. An analogous protocol was established for patient 1. After the reduction, a constant frequency reduction was performed followed by a total of five clinical stress tests (A, B, C, D, E). For tests B and C, a slightly extended pump stop time of 10 min was used. For the last 2 tests, a 30 min pump stop time was used. The weaning process was carried out over several weeks.

## 3. Case Studies—Detailed Presentation

### 3.1. Case Report 1

**Figure 1:**
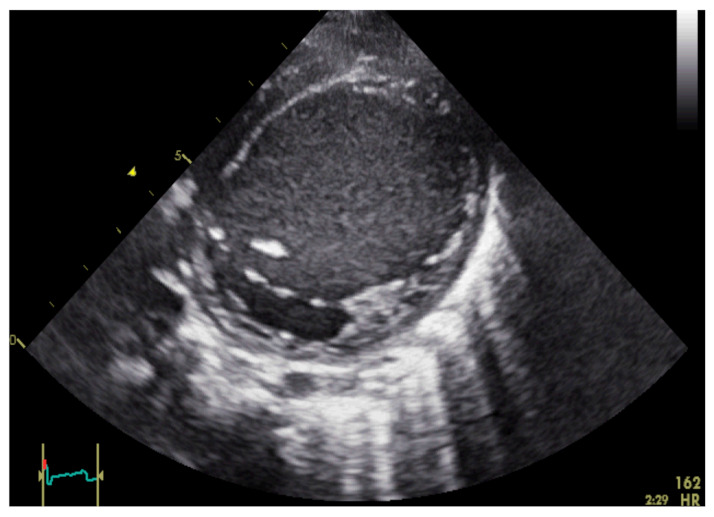
Patient 1 short axis view upon arrival at the clinic, showing an enlarged left ventricle with a thin wall and spontaneous contrast within the left ventricular cavity.

A 8-month-old girl presented with massively impaired left ventricular function (EF 35%, LVEDD 44 mm (+2z), aortic VTI 6.5 cm at HR 156/min, <Pc 3, ST: global longitudinal strain [GLS] 4Ch −6.5%, 2Ch −6.4%, Figure 1). The girl showed the progressive deterioration of her general condition within two weeks with feeding intolerance, tachydyspnoea and diarrhoea. The symptoms led to repeated visits to the family doctor’s office, and the girl was admitted to the paediatric ward of a paediatric and adolescent medicine clinic. Shortly after admission, she was referred to our tertiary cardiac centre with the suspicion of acute myocarditis and the phenotype of dilated cardiomyopathy. Up to this point, no other diseases were diagnosed, and she showed normal somatic as well as neurological development. Family history was negative, and there was no consanguinity. She had a healthy 5-year-old brother.

The biopsy (EMB) revealed histological diagnosis of chronic myocarditis, DD (differential diagnosis) of dilated cardiomyopathy with mild fibrosis. Metabolic disease and coronary anomaly were excluded. Neither family history nor genetic testing revealed any evidence of major known cardiomyopathies (testing of about 147 gens). Implantation was performed on the fourth day after admission due to further clinical deterioration and ventricular tachycardia. A 15 mL ventricle was used. Postoperative catecholamine therapy was discontinued within three weeks. A weaning schedule was followed for 14 weeks. The program started with a daily reduction of the Berlin heart rate (BH) for 2 h/day to 50/min for three weeks (phase A), an extension to 4 h/day for a fortnight and a complete reduction to 50/min for nine weeks (phase B). To test myocardial recovery, a 30-min pump stop was performed once a week to assess clinical improvement under additional heparin bolus (phase C). All steps were monitored by echocardiographic measurements. Before explantation, a catheter examination was performed to confirm the echocardiographic data. The initial training phase (phase A) resulted in an improvement in strain scores from −8% (GLS 2Ch) and −10% (GLS 4Ch) to results of −13% and −11%, respectively, at the end of the training phase. VTIs were initially measured between the 3rd and 10th Pc and increased up to the 10th Pc. [8]. The prolongation of daily reduction showed a slight increase in LVEDV from 55 mL/m^2^ to 57 mL/m^2^ (>97th Pc) at the beginning, but it remained constant at 59 mL/m^2^ at the end of the 2-week training phase. VTI also remained at the 10th Pc and ejection fraction increased from EF 32% to 36%. In phase C, parameters were obtained during pump stop. Data were collected every 5 min and post-processed. An increase in VTIs and EF was observed, as well as a reasonable adjustment in stroke volume without an increase in left ventricular volume. VTIs were above the 90th percentile. The exercise data showed a slight improvement. A test is shown in the attached Table 1.

**Figure 2:**
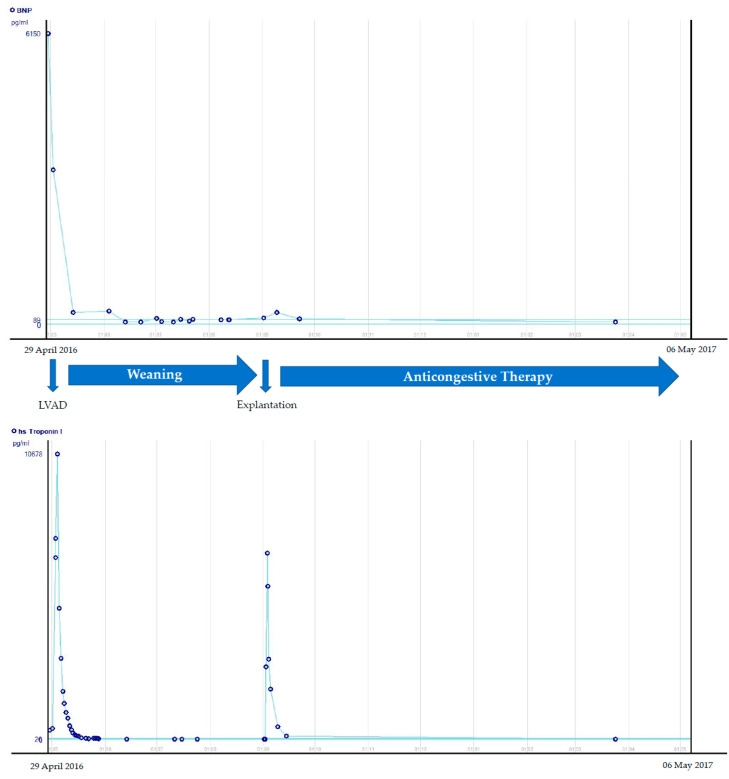
Development of BNP and troponin during weaning and afterwards in Patient 1.

The final haemodynamic examination in the catheter laboratory revealed no relevant changes in the parameters: on Berlin Heart (BH) 50/min: CI (cardiac index) 4.1 L/min/m^2^, CVS (central venous saturation) 60%, LVEDP (left ventricular enddiastolic pressure) 8 mmHg. Pump stopped for 30 min: CI 3.6 L/min/m^2^, CVS 58%, LVEDP 10 mmHg. The Development of the laboratory parameters during the weaning process is plotted over time (Figure 2).

The device was successfully removed, and the patient was treated with standard congestion therapy. She visits our outpatient clinic regularly, and left ventricular function has now been stable for over six years, with normal heart function and no medical therapy.

### 3.2. Case Report 2

An 18-month-old female patient presented to us with suspected acute myocarditis/DD dilated cardiomyopathy. The family history was negative. She was the third child in the family. She had been ill with hand, foot and mouth disease three weeks previously and had received symptomatic supportive care with no further problems. On admission, the child’s condition was severely compromised. We saw a massively dilated left ventricle (LVEDV 190 mL/m^2^, LVESV 136 mL/m^2^, EF 30%, SV 23 mL) Despite intensive inotropic support, the rapid progression of heart failure was noted, and the implantation of an LVAD was performed two days after hospital admission. Family genetic testing revealed no abnormalities. The pump ventricles had a volume of 15 mL with an initial rate of 100/min (CI 3 L/min/m^2^). Molecular pathological diagnosis revealed acute lymphocytic parvovirus B 19 myocarditis. Postoperative catecholamine therapy was discontinued within four weeks. Thereafter, the baseline pump rate was reduced to 85/min (CI 2.5 L/min/m^2^). At this point, the weaning plan could be implemented. The weaning plan was implemented over 19 weeks. We started in this case with a daily reduction in rate for 4 hrs/day to 70/min for 5 weeks; a reduction in base rate to 75/min daily and a reduction from 4 hrs/day to 60/min for 4 weeks (phase A); complete reduction in base rate to 60 bpm (CI: 1.8 L/min/m^2^) for further 2 weeks (phase B). To check cardiac improvement, a 30 min pump stop was performed once a week (phase C). All steps were carefully documented echocardiographically.

The first 5 weeks showed a progressive reduction in LVEDV from 190 mL/m^2^ to 100 mL/m^2^ and an increase in stroke volume. Deformation evidenced by ST data remained modest, with overall values of −10%. There was no change in vital signs during the pump stop tests. BGAs and lactate production were normal. The aortic VTIs remained consistently above the 90th Pc. The final pump stop test showed an LVEDV of 96 mL/m^2^ (>97th Pc), an ESV of 55 mL/m^2^, an SV of 21 mL and an EF of 47%. During pump stop, there was an increase in VTIs and EF and an adequate adjustment in stroke volume without an increase in LVEDV.

ST data demonstrated a slight improvement after ten weeks of participation in the program. However, deformation was not completely normal; some wall motion abnormalities were still persistent. The explantation of the device was successful; the patient could be weaned off the catecholamines and was treated with standard anticongestive therapy. One month after explantation, echocardiographic examination was excellent (LVEDV 89 mL/m^2^, LVESV 50 mL/m^2^, EF 46%, SV 21.3 mL, HR 100/min, CI 4.5 L/min/m^2^, VTI aortal 16 cm (50th pc)). The patient has been managed in our outpatient clinic for four years since explantation, with normalised cardiac function and no medication.

### 3.3. Case Report 3

**Figure 3:**
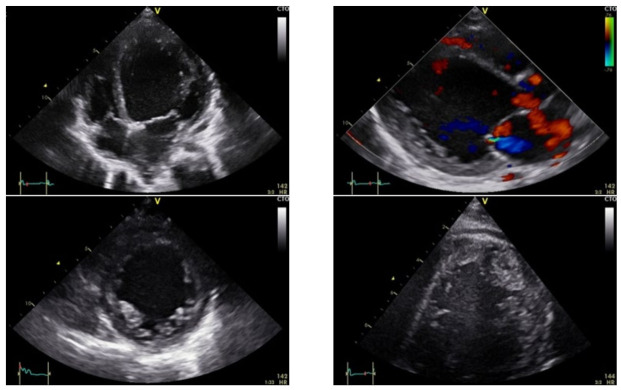
Patient 3: Four-chamber view, long axis and short axis on admission to hospital showing mitral regurgitation and severe trabeculation of the left ventricle. Blood clots may be suspected at the apex.

**Figure 4:**
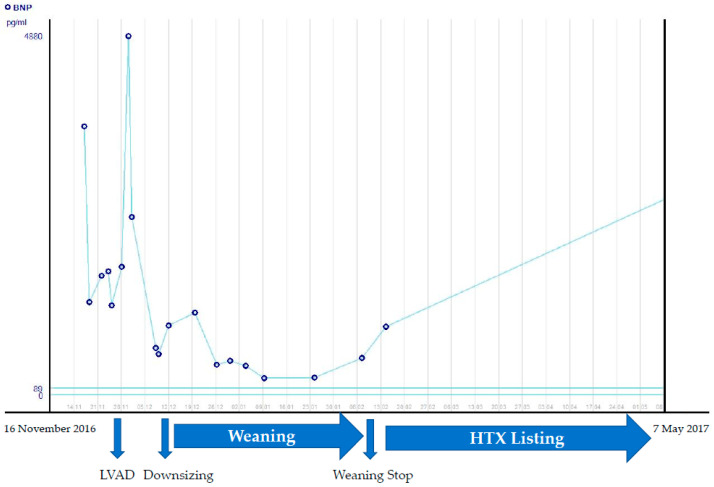
Development of BNP in Patient 3.

A female patient aged 8 5/12 years was referred from a paediatric clinic with suspected acute myocarditis. She presented with fever, vomiting and dehydration with ketoacidosis for one week. Admission to the paediatric ward and specific therapy followed. During hospitalisation, she developed a cough and underwent a chest X-ray that revealed cardiomegaly, leading to a further diagnosis. Family history revealed a paternal grandfather who died of heart disease at the age of 54. The patient’s mother was diagnosed with ventricular intermittent arrhythmia during the patient’s hospitalisation. On closer cardiological examination, we found that the mother had mild dilated cardiomyopathy with noncompaction of the left ventricle. The patient’s cardiac function was massively impaired (EF 20% LVEDD 59 mm (+3.6 z), aortic VTI 13 cm at 130/min, 25–50th Pc). Deformation parameters were pathological: ST GLS 4Ch −5%, 2Ch −5%. RT3DE showed LVEDV of 140 mL/m^2^ (>97Pc), LVESV of 104 mL/m^2^, SV of 28 mL, EF 20% at a HF of 140/min–CI 3.6 L/min/m^2^. As shown in Figure 3, there were abnormal trabeculations of the left ventricle—especially at the apex—and the heart was massively enlarged. The echocardiographic aspect was typical for a noncompaction disorder (LVNC) with DCM. A clinical assessment of the patient at this time did not reveal any relevant restrictive dysfunction (tissue Doppler- E/E′ medial 10, lateral 7, TDI S/E′/A′ 4/7/9 cm/sec- medial, 8/9/4 cm/sec- lateral). She was initially stabilised with catecholamine therapy (dobutamine, milrinone, levosimendan) and diuretics. Eight days after admission to hospital, LVAD implantation was decided due to resuscitation for ventricular fibrillation. This case involved a 30 mL Berlin Heart LVAD with an initial rate of 100/min. Postoperative catecholamine therapy was rapidly reduced, and the ventricle recovered unexpectedly, causing filling problems even though we reduced the rate to a minimum and discontinued most of the inotropic drugs for the right ventricle. The risk of blood clots was immense. On the 15th day after implantation, a ventricular replacement was forced due to thrombus formation. Due to the short recurrence of clot formation in the inflow cannula, downsizing to a 15 mL ventricle was necessary (15 mL at a rate of 100/min). Thus, a reduction of the secured CI from 1.8 L/min/m^2^ to 1.5 mL/min/m^2^ was performed. Biopsy revealed a diagnosis of mild chronic active/DD in healing HHV6 (human herpesvirus 6) myocarditis with diffuse perivascular fibrosis. Histology of the biopsy did not show unequivocal evidence for the diagnosis of dilated cardiomyopathy. Genetic examination of the patient did not reveal any proven genetic alteration that could explain the clinical picture. The molecular diagnosis showed the persistence of a low copy number of HHV6 variant B. This was not consistent with the serological findings. Probational ganciclovir therapy was discontinued after two weeks because of the patient’s neurological impairment with seizures. Acute myocarditis was ruled out. CT and EEG showed no evidence of major cerebral lesions. The weaning protocol was implemented. It differed from that of the other two patients in that the ventricle was rapidly reduced in size. Practically, it comprised two phases, the first immediately after reduction (still on catecholamine therapy) and the second with a constant reduced rate of 80/min. The start of the second phase was decided within four weeks after implantation, after the cessation of catecholamine therapy. Analogous to the other two patients, the tests were performed with a pump stop under heparin protection. In total there were 5 pump-down tests in 8 weeks. The first test took place one week after ventricular reduction and after the cessation of catecholamine therapy and showed promising results (Table 2). A slightly prolonged pump stop time of 10 min was used in the 2nd and 3rd tests. Ejection fraction, aortic VTI and RT3DE LVEDV showed no relevant changes. A 30 min time window was used for the last 2 stops. Both resulted in an increase in LVEDV assessed with RT3DE from an on-pump volume of 140 mL/m^2^ to 155 mL/m^2^ and inadequate stroke volume adjustment (Table 3). Even though the initial results were promising, recovery could not be accomplished. The echocardiographic parameters deteriorated. We saw unfavourable development of cardiac geometry and deterioration of cardiac function with reduced EF. The BNP also showed an increasing trend (Figure 4). The girl was put on the HTX (high emergency transplant) list and received an organ within a short waiting period.

## 4. Discussion

This new protocol not only enables the identification of recovery candidates, it could also enable recovery. Long-term survival after weaning seems to be better than after transplantation [10]. Therefore, it is particularly important not only to identify candidates for weaning but to encourage and support the process.

### 4.1. Practical Approach

Common to all three protocols was the progressive loading of the ventricle over a patient-adapted period of time under noninvasive (clinical and echocardiographic) assessment and modification of support as needed based on changes in the heart. McCarthy et al. showed that ventricular function may well recover after acute fulminant myocarditis [11]. Levin Gr et al. postulated that unloading of the venous ventricle using mechanical support (“empty beating heart” phase) influences several neurohormonal cardiovascular factors, leading to the normalisation of venous geometry. The authors referred to this process as “reversible remodelling”, which leads to an improvement in function [12]. One of the meticulously documented parameters for geometry was LVEDV. An increase in volume was interpreted as decompensation rather than improvement of the ventricle. Cardiac function was monitored with several parameters. In addition to the standard values for cardiac function such as EF, VTIs were an important decision factor. In both patients, the percentiles were above 90th Pc. Thus, the protocol was continued with increasing the mechanical load on the ventricle by reducing the pump rate daily and, if tolerated, for several weeks. In the first two patients, the deformation was not optimal and improved only slightly. The ventricular cannula changes the motility and geometry of the ventricle. Normal deformation is not a realistic goal. The myocardium still has many intrinsic or iatrogenically caused scar regions.

However, there are some echocardiographic measurements that have been shown to be very informative for long-term weaning. [2,13,14,15]. The main expertise in weaning has been achieved in adult patients with chronic heart failure and mostly with nonpulsatile devices. The strategy varies from institution to institution, from pharmacological intervention (with high doses of congestive medications) followed by pump stop testing [14] to dobutamine testing and the assessment of left ventricular wall thickness during development [15]. In the adult population, the physical stress test is also commonly performed. However, in the paediatric population it is almost impossible to obtain the equivalent of a 6-min walk test. The ages of patients 1 and 2 clearly demonstrate this. Therefore, the paediatric cardiologist is forced to use innovative methods to test the ventricular load capacity.

The factors that influenced the decision to leave the patients on the protocol were: a preserved sinus rhythm and the RT3DE LVEDV, SV and EF measurements. The deformation parameters are also important but as a long-term observation rather than an acute decision aid.

For rapidly increasing volume, we modified the protocol as needed with a slower reduction in pump rate or reduction interval. Repeated reduction in stroke volume associated with an increase in left ventricular volume was a prognostic negative sign of failed adaptation of the ventricle to the patient’s needs. The nonresponder showed an increase in LVEDV of about 10% and a relevant decrease in stroke volume and EF in the last two tests. Based on this experience, we have introduced a new protocol for ventricular training and weaning for these challenging cases in our centre (Table 4).

### 4.2. Biopsy

Our experience has shown the importance of ruling out major metabolic causes of dilated cardiomyopathy (DCM) or genetic causes. According to a review of the literature, the total annual incidence of cardiomyopathies in childhood is estimated at 1:100,000 [16]. There are very few large prospective studies. The majority of publications report retrospective data. The incidence for primary forms of CMP is thought to be highest at a young age [16,17]. According to the subgroup classification of cardiomyopathies, most publications report an incidence of DCM of up to 51–60%. In up to one-third of DCM, signs of myocarditis have been detected on biopsy [18]. In addition, the DCM phenotype is observed in nearly 50% of myocarditis in infants under two years of age [19,20].

Hypertrophic cardiomyopathies are the second most common form with an incidence of 25–40%. Many children have a mixed phenotype or undergo transition during the course of the disease. Reported incidence may vary by region, race or ethnicity and diagnostic capabilities [16,18,21].

Children in post-myocarditis status are expected to have a better chance of recovery of function [10]. Acute myocarditis in children often takes a fulminant course [22]. Myocarditis is an inflammatory disease of the myocardium associated with necrosis, apoptosis, fibrosis and, in the recovery phase, remodelling [23,24]. Myocarditis can be caused by viral (adenoviruses, echoviruses, enteroviruses, herpes viruses, e.g., CMV, EBV, HHV6; hepatitis C; human immunodeficiency virus; influenza type A; and parvovirus B19) or nonviral infections (chlamydia, mycoplasma, legionella, streptococcus, fungal infections, protozoa, spirochete or rickettsia). Less commonly, it is the result of an autoimmune reaction or can be triggered by a hypersensitivity reaction to drugs or vaccines [25].

A multicentre prospective registry on myocarditis in paediatric patients aims to gain insights into its manifestation, diagnosis and optimal therapy [19].

The currently published data from the registry confirm based on histopathological analysis an inflammatory process in 73% of the included patients. Viral detection (PCR in myocardium or blood sample) may depend on the time of diagnosis. The possibility of the regression of fibrosis and reduction of inflammation are prognostically important [20].

Myocarditis may progress to dilated cardiomyopathy. Many publications describe dilated cardiomyopathy as the most common cause of heart transplantation [25,26]. The cases in which the clinical situation deteriorates rapidly despite medical treatment require mechanical circulatory support as a bridge to recovery or as a bridge to transplantation. Decision-making and prognosis are often influenced in part by myocardial biopsy results. The Dallas criteria have been widely used in histological diagnosis since 1987. In addition, the 2013 European Society of Cardiology (ESC) Task Force consensus document recommends the collection of endomyocardial biopsies (EMB) in patients with clinical suspicion of myocarditis. EMB is still considered the gold standard for diagnosis. [27]. It is based on conventional histology (Dallas criteria) and immunohistochemistry and PCR of infectious agents. Nevertheless, there is a high variance between servers [25].

The EMB was obtained intraoperatively in all three of our patients at the time of implantation of the Berlin heart (BH) left ventricular assist device (LVAD). Patient 1 was diagnosed with dilated cardiomyopathy with chronic myocarditis. No. 2 tested positive for parvovirus B19, a clear case of acute viral myocarditis. In patient 3, the EMB result was inconclusive. The primary echocardiographic diagnosis and macroscopic surgical suspicion in patient 3 was that of possible decompensated dilated noncompaction cardiomyopathy. In the literature, we were able to find a number of case reports on the histopathological changes in noncompaction cardiomyopathy [28]. All publications show mostly marked fibrosis. McMahon and Pignatelli, in their publications on noncompaction cardiomyopathy in children, postulated that there are “undulating phenotypes” of LVNC with the transition between dilated and hypertrophic phenotypes—patients show improvement followed by deterioration over time [29]. The fact that the mother of patient 3 most likely also suffers from dilated cardiomyopathy is highly suspicious, but the genetic examination could not detect a pathological genetic change according to the current state of the art.

### 4.3. Genetics

Genetic predisposition is an important aspect that scientists are looking at intensively today. In a 1994 publication, Caforio showed organ-specific cardiac antibodies in 342 relatives of DCM patients as a sign that the immune system is a very important regulator of the way cells respond to injury [12,30]. In a later publication in 2008, the same author concluded that myocarditis and DCM in large populations are just different stages of chronic, mostly autoimmune, damage to the heart muscle. The process is thought to be at least to some degree genetically determined [31]. The hypothesis is that some cases of dilated cardiomyopathy and myocarditis are influenced by a genetic predisposition. We performed genetic testing in these three patients and could not find any clear pathological genetic alteration. The interesting result is that patient 1 had no suspicious variants and patient 2 had only ACMG-3 variants in the panel sequencing. Patient 3 also had only ACMG-3 variants, but as the mother also had DCM, we suspect that a gene variant not included in the sequencing may be involved. We would recommend genetic testing for any patient with suspected cardiomyopathy or myocarditis. Severe genetic alterations can be an important deciding factor for the further course of therapy. Similarly, the absence of a genetic change may raise hope that the process is reversible.

## 5. Limitations

The predictive factors for successful weaning are not very well known, let alone well defined. A larger number of patients is needed to identify the risk factors and define the weaning potential. In addition to initial volume offloading, decreased fibrosis development and inflammation and a favourable genetic constellation are prognostically important for the onset of progressive reloading and exercise. Patients 2 and 3 had genetic modifications of unknown significance. These modifications may nevertheless have a phenotypic expression that affects outcome and therapeutic decision-making. Knowledge about the phenotypic expression of certain genetic modifications is still limited. The heterogeneity of patients is certainly also a limitation. The weaning process represents an idea that must be tailored to the individual needs of the patient and continuously adapted to the new clinical data and haemodynamic changes.

## 6. Conclusions

Retraining a weakened heart may be a viable option for patients with heart failure on VAD therapy. To achieve the best prognosis, early support is essential to avoid prolonged periods of low cardiac output, end-organ dysfunction or cardiac arrest [3,32]. Once the patient has been stabilised, individualised protocols should be established, tailored precisely to the patient’s cardiac and clinical situation, even after prolonged VAD therapy. This requires a team of experienced physicians with good clinical experience, comprehensive knowledge of haemodynamics and a high-quality standard of echocardiographic examination. New echocardiographic tools and an individualised weaning protocols can help assess improvement in function, which affects prognosis. In the paediatric group without obvious metabolic disease, it is important to stress the damaged muscle to potentially restore cardiac function.

## Figures and Tables

**Table 1:** Pump stop parameters of patient 1.

Measurements Phase C	Pump On	Pump Off 15 min	Pump Off 30 min
HR	120	124	127
VTI pulmonary	12 cm	14 cm	17 cm
VTI aortic	17 cm	17 cm	18 cm
LVEDV	79 mL/m^2^		89 mL/m^2^
LV-SV	15 mL		17 mL
EF	47%		50%
GLS4Ch	−9.5%	−12%	−13.5%
GLS2Ch	−13.6%	−13%	−15%

**Table 2:** Weaning Test A-Patient 3.

HR	100/min	50/min	Pump Stop 5 min
VTI aortal	10 cm	15–20 cm	20 cm at 100/min
EF Simpson	30%	25%	30%
LVEDD	47 mm (+1.3 Z)	42 mm (+0.23 Z)	51 mm (+2.8 Z)
LVEDV RT3DE	88 mL/m^2^		100 mL/m^2^
GLS4Ch	−6%		−6%

**Table 3:** Weaning Test E-Patient 3.

HR	80/min	30 min Pump Stop
**VTI aortal**	16.5 cm	18 cm at 104/min (25th Pc)
**LV EDV** **RT3DST**	140 mL/m^2^	155 mL/m^2^
**EF**	25%	15%
**GLS4Ch**	−3%	−3%
**LVSV**	35	37

**Table 4:** New algorithm for cardiac muscle training.

Echocardiographic Parameters (Echo)	Left ventricular end-diastolic diameter (LVEDD)
	LV EF, optional RV- EF
	Velocity time integral aortal and pulmonal (VTI’s)
	LVEDV, LVESV-RT3DE
	dp/dt MI (Mitral insufficiency), TAPSE
	Deformation parameter with speckle tracking method (ST)
Blood work-up once a week	BNP, Troponin, GOT, GPT, Creatinin, ABG
ECG	Sinus rhythm (SR)
Normal cardiac index under LVAD (>2 L/min/m^2^ BSA)	1. Temporary reduction of pump frequency by 10/min for 4 h. Echo before an the end of testing 2. After at least 3 test of temporary reduction of frequency and stable conditions, permament reduction by 10/min if tolerated without increase of volumes more than 20%. 3. Repeat steps 1 and 2 until residual frequency is 10 beats higher than minimal sugested LVAD frequency by the manufacturer 4. Three separate pump stops under heparin bolus (100 Units/kg) and inermittend hand pump with low frequency for 30 min. Echo at baseline and after 10, 20 and 30 min. ABG before an and the end of the testing and noninvasive bloodpressure monitorig. If central line then central venous pressure monitorig. CAVE: be carefull to maintain the same measured parameter in order to have a feasible comparison from stage to stage. 5. If cardiac output is stable with normal cardiac index, more than 40% systolic thickening in MMODE, LVEDV <97th Pc. and EF >50%: go to explantation with final testing in OR.

## Data Availability

Not applicable.
